# Alkannin Inhibits the Development of Ovarian Cancer by Affecting miR-4461

**DOI:** 10.1155/2021/5083302

**Published:** 2021-11-28

**Authors:** Yaowen Wang, Jingfang Zhang, Feipeng Wang, Wenping Chen, Jie Ma, Honggang Wang

**Affiliations:** ^1^Department of Clinical Laboratory, Weifang People's Hospital, Weifang 261041, Shandong Province, China; ^2^Department of Gynaecology, Linyi County People's Hospital, Dezhou 251500, Shandong Province, China; ^3^Department of Radiophysics, Affiliated Qingdao Central Hospital, Qingdao University, Qingdao 266042, Shandong Province, China; ^4^Department of Cardiothoracic Surgery, The People's Hospital of Zhangqiu Area, Jinan 250200, Shandong Province, China

## Abstract

**Background:**

Previous studies have shown that alkannin has anticancer, anti-inflammatory, and antibacterial effects. However, the effect of alkannin in the development of ovarian cancer (OC) remains unknown. Therefore, this study aims to elucidate the function of alkannin in OC progression.

**Methods:**

RT-qPCR and western blot analysis were used to measure mRNA and protein expression. Cell viability and metastasis were detected by the CCK-8 assay, flow cytometry analysis, and transwell assay.

**Results:**

Alkannin had no cytotoxicity toward normal ovarian cells, but alkannin can inhibit cell proliferation and induce apoptosis in OC cells. In addition, alkannin inhibited cell migration and invasion and blocked EMT in OC. Besides, upregulation of miR-4461 was found in OC tissues and cells, which was regulated by alkannin. More importantly, miR-4461 can inverse the effects of alkannin on cell viability and metastasis in OC cells.

**Conclusion:**

Alkannin restrains cell viability, metastasis, and EMT in OC by downregulating miR-4461 expression.

## 1. Introduction

Ovarian cancer (OC) is one of the common malignant tumors of female reproductive organs, and its incidence is second only to cervical cancer and uterine body cancer [1]. OC is the most common epithelial cancer, followed by malignant germ cell tumors. Among them, the mortality rate of epithelial OC ranks first among all kinds of gynecological tumors [2], which can pose a serious threat to women's lives. OC is mostly asymptomatic in the early stage, and gastrointestinal symptoms such as lower abdominal discomfort, bloating, and loss of appetite may appear at the advanced stage [3]. The main treatment methods for OC include surgical resection, drug therapy, and radiation therapy. The overall prognosis of OC patients is poor, and OC is generally difficult to cure [4, 5]. The 5-year survival rate of early patients is higher. However, advanced patients are prone to recurrence and metastasis. Some patients will experience adverse reactions after radiotherapy [6]. Therefore, it is necessary to find effective drugs with low side effects to treat OC.

Alkannin is a crystalline powder found in the roots of *Lithospermum erythrorhizon* Sieb. et Zucc. and *Arnebia euchroma* (Royle) Johnst [7]. Alkannin is often used to treat acute icteric or nonicteric hepatitis, chronic hepatitis, and flat warts [8]. In addition, alkannin is also effective for liver cirrhosis (ascites) and common warts [9]. Besides, alkannin has been reported to regulate tumorigenesis. For example, alkannin inhibited the growth and invasion of C6 glioma cells through the IQGAP/mTOR signaling pathway [10]. Alkannin restrained the growth, migration, and invasion of oral squamous carcinoma cells by regulating the microRNA-9/RECK axis [11]. However, the function of alkannin in the progression of OC has not been reported.

Many studies have shown that traditional Chinese medicine can regulate the development of human cancer by mediating miRNAs [12, 13]. For example, apigenin suppressed the proliferation, invasion, and epithelial-mesenchymal transition (EMT) of cervical carcinoma cells by regulating the miR-152/BRD4 axis [14]. Curcumin inhibited prostate cancer progression by regulating the miR-30a-5p/PCLAF axis [15]. In addition, alkannin has been found to inhibit the growth of pancreatic cancer cells by downregulating miR-199a [16]. Here, the interaction between alkannin and miR-4461 was investigated in OC. miR-4461 has been reported to inhibit the tumorigenesis of renal cell carcinoma by targeting PPP1R3C [17]. However, miR-4461 was regarded as a potential onco-miRNA in OC by targeting PTEN [18]. These results indicate that miR-4461 is tissue specific.

In this study, the effects of alkannin on the viability and metastasis of OC cells were observed. And the regulatory mechanism of alkannin and miR-4461 was preliminarily discussed. This research lays an experimental and theoretical basis for the clinical treatment of OC.

## 2. Materials and Methods

### 2.1. Clinical Tissues

Forty-eight OC patients from Weifang People's Hospital participated in the study. Before the start of the experiment, we obtained informed consent of all OC patients. Except for surgery, OC patients did not receive any treatment. This study has been approved by the Ethics Committee of Weifang People's Hospital.

### 2.2. Cell Culture

Ovarian epithelial cells (HOSEpiC) or OC cell line SKOV3 were purchased from ATCC (Manassas, VA, USA). The growth conditions are 5% CO_2_, 37°C, and culture medium (90% RPMI-1640 + 10% FBS).

### 2.3. Alkannin Treatment

Alkannin (purity>95.0%, Sigma, St. Louis, MO, USA) was dissolved in methanol and diluted in the RPMI-1640 medium to concentrations of 0, 1, 5, 10, 15, and 20 *μ*M. HOSEpiC and SKOV3 cells were incubated with different doses of alkannin for 12 h. SKOV3 cells were treated with 10 *μ*M alkannin for 0, 2, 4, 6, 8, 10, 12, 14, and 16 h to detect the effects of alkannin treatment time.

### 2.4. Cell Transfection

miR-4461 mimics and inhibitor were synthesized by RiboBio (Guangzhou, China). They were transfected into alkannin-treated SKOV3 cells using Lipofectamine 2000 (Invitrogen, Carlsbad, USA), respectively.

### 2.5. RT-qPCR

Total RNA extraction was performed using TRIzol reagent (Invitrogen). The cDNA solution was synthesized using the miScript II RT Kit (Qiagen, Valencia, CA, USA). RT-qPCR was performed on an ABI 7500 Fast Real-Time PCR system using miScript SYBR Green PCR Kit (Qiagen). miR-4461 was normalized to the U6 internal reference. The primer sequences used were as follows: miR-4461, forward: 5′-GAG TGTCGGGGCAGCTCAGT-3′ and reverse: 5′-GCAGGGTCCGAGGTATTC-3′; U6, forward: 5′-GCTTCGGCAGCACATATACTAAAAT-3′ and reverse: 5′-CGCTTCACGAATTTGCGTGTCAT-3′. Their expressions were quantified using the 2^−△△ct^ method.

### 2.6. CCK-8 Assay

SKOV3 cells were added in a 96-well plate and incubated for 12 h. Then, the culture was added with different concentrations of alkannin and incubated for 0, 2, 4, 6, 8, 10, 12, 14, and 16 h. Next, 10 *μ*l of CCK-8 solution was added to incubate these cells for 1 h. Finally, the wavelength of 450 nm in each well was measured on a Microplate Reader (ELx800, BioTek Instruments, Winooski, VT).

### 2.7. Flow Cytometry Analysis

The apoptosis of SKOV3 cells was detected after treatment with alkannin (10 *μ*M) for 24 h. Following the instructions of Annexin V-Phycoerythrin (PE) Kit (Beyotime Biotechnology, Shanghai, China), SKOV3 cells were washed with PBS. After discarding the supernatant, the cells were resuspended in 100 *μ*L of binding buffer. 4 *μ*L Annexin V-FITC and 5 *μ*L PI staining solution were added to each tube. After 15 minutes of incubation in the dark, the apoptosis rate was detected by flow cytometry.

### 2.8. Transwell Assay

The membrane on the bottom of the transwell was coated with Matrigel. The cells were adjusted to a density of 5 × 10^5^ cells/mL with serum-free medium. 200 *μ*L of cell suspension was added to the upper chamber of the transwell. 500 *μ*L of the culture medium containing 20% FBS was added to the lower chamber. The cells were cultured for another 12 h, and the transwell chamber was taken out and washed twice with PBS. The upper chamber cells were wiped with cotton swabs, fixed with methanol for 15 min, and stained with 0.1% crystal violet at room temperature for 30 min. The number of invaded cells was counted under the microscope. Matrigel is not used in the migration experiment, and the rest of the steps are the same as the invasion experiment.

### 2.9. Western Blot Analysis

RIPA lysis buffer was used to obtain protein samples. Next, 10% SDS-PAGE was used to separate 25 *μ*g of protein. Protein samples were transferred to the PVDF membrane. Blocked with 5% skimmed milk, the protein samples were incubated with vimentin, E-cadherin, N-cadherin, and GAPDH primary antibodies (Abcam, Cambridge, MA, USA) at 4°C overnight. Then, protein samples were incubated with HRP-conjugated secondary antibody (Abcam, USA) for 1 h. Finally, ECL (ECL, Pierce) was used to measure protein expression. Quantity One 4.52 analysis software was used to measure the gray value of the band. Relative expression of target protein (IOD) = gray value of the target protein/gray value of internal reference GAPDH.

### 2.10. Statistical Analysis

All experiments were repeated three times. Data are expressed as mean ± SD and analyzed by SPSS 18.0 or GraphPad Prism 6. One-way ANOVA with Bonferroni post hoc test was used to analyze differences between groups. *P* < 0.05 is considered as a statistically significant difference.

## 3. Results

### 3.1. Alkannin Inhibits Cell Proliferation and Induces Apoptosis in OC

**Figure 1:**
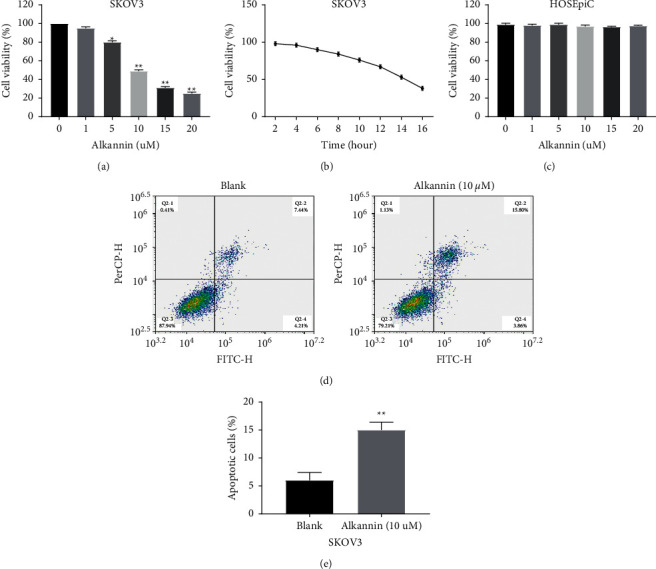
Alkannin inhibits cell proliferation and induces apoptosis in OC. (a) The viability of SKOV3 cells treated by alkannin at concentrations of 0, 1, 5, 10, 15, and 20 *μ*M for 12 h was detected. (b) SKOV3 cells were treated by 10 *μ*M alkannin for 0, 2, 4, 6, 8, 10, 12, 14, and 16 h. (c) The viability of ovarian epithelial cells HOSEpiC treated by alkannin at concentrations of 0, 1, 5, 10, 15, and 20 *μ*M for 12 h was detected. (d) Cell apoptosis was detected in SKOV3 cells treated by alkannin (10 *μ*M) for 12 h. ^*∗*^*P* < 0.05 and ^*∗∗*^*P* < 0.01.

In order to explore the effect of alkannin on the viability of OC cells, CCK-8 assay was performed. The results showed that alkannin at concentrations of 5 (*P* < 0.05), 10 (*P* < 0.01), 15 (*P* < 0.01), and 20 *μ*M (*P* < 0.01) suppressed cell proliferation in SKOV3 cells (Figure 1(a)). Because the 50% inhibitory effect of alkannin on cell viability appears at about 10 *μ*M, therefore, 10 *μ*M alkannin was used for the following experiments. Next, we found that the decline in SKOV3 viability caused by alkannin appeared in a time-dependent pattern. The best-fit value for the variable slope is approximately 12 h (Figure 1(b)). Hence, SKOV3 cells were treated with 10 *μ*M alkannin for 12 h in the following experiments. In addition, the viability of ovarian epithelial cells HOSEpiC after treatment with 0, 1, 5, 10, 15, and 20 *μ*M alkannin was detected. We found that the cell viability of HOSEpiC cells did not change significantly (*P* > 0.05, Figure 1(c)). The result indicates that alkannin is not cytotoxic to normal ovarian cells. Moreover, flow cytometry analysis showed that alkannin significantly induced apoptosis (*P* < 0.01, Figure 1(d)). All these results demonstrate that alkannin can inhibit cell proliferation and induce apoptosis in OC.

### 3.2. Alkannin Inhibits the Migration and Invasion of OC Cells

**Figure 2:**
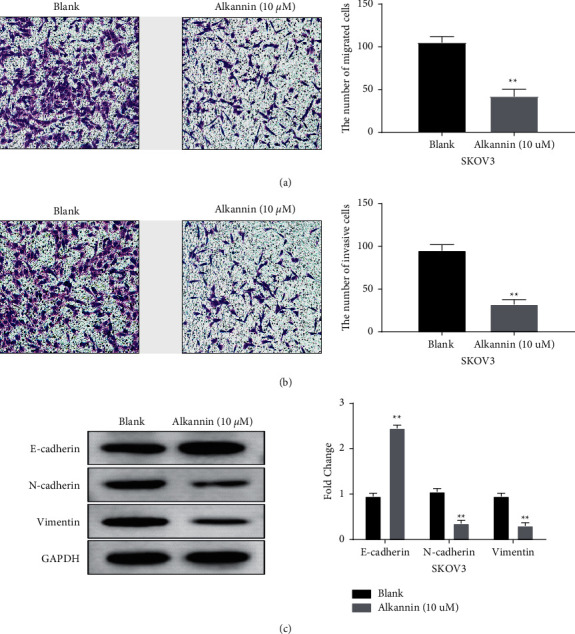
Alkannin inhibits the migration and invasion of OC cells. (a, b) Cell migration and invasion were detected in SKOV3 cells treated by alkannin (10 *μ*M) for 12 h (magnification, 200x). (c) The expression of E-cadherin, N-cadherin, and vimentin was measured in SKOV3 cells treated by alkannin (10 *μ*M) for 12 h ^*∗∗*^*P* < 0.01.

To further explore the function of alkannin in OC, transwell assay was performed to measure cell migration and invasion. We found that alkannin inhibited cell migration in SKOV3 cells (*P* < 0.01, Figure 2(a)). Moreover, the inhibitory effect of alkannin on cell invasion was also found in SKOV3 cells (*P* < 0.01, Figure 2(b)). In addition, the effect of alkannin on EMT was also investigated in SKOV3 cells. Western blot analysis showed that alkannin enhanced the expression level of E-cadherin and inhibited N-cadherin and vimentin expressions in SKOV3 cells (*P* < 0.01, Figure 2(c)). The above results indicate that alkannin inhibits cell metastasis and blocks EMT in OC.

### 3.3. miR-4461 Expression Is Upregulated in OC Tissues and Cells

**Figure 3:**
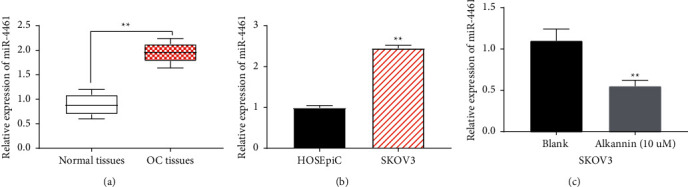
miR-4461 expression is upregulated in OC tissues and cells. (a) The expression level of miR-4461 was determined by RT-qPCR in OC tissues and normal tissues (*n* = 48). (b) miR-4461 expression was detected in HOSEpiC and SKOV3 cells. (c) miR-4461 expression was assessed in SKOV3 cells treated by alkannin (10 *μ*M) for 12 h ^*∗∗*^*P* < 0.01.

In order to confirm whether miR-4461 is involved in the progression of OC, the expression of miR-4461 in OC tissues and cells was evaluated. RT-qPCR showed that miR-4461 expression in OC tissues was higher than that in normal tissues (*P* < 0.01, Figure 3(a)). Compared with ovarian epithelial cells HOSEpiC, miR-4461 in SKOV3 cells was upregulated (*P* < 0.01, Figure 3(b)). In addition, we found that miR-4461 expression in SKOV3 cells treated with alkannin was significantly reduced (*P* < 0.01, Figure 3(c)). These findings confirm the abnormal expression of miR-4461 in OC tissues and cells, indicating that miR-4461 may play an important role in the pathogenesis of OC.

### 3.4. Alkannin Restrains the Progression of OC by Decreasing miR-4461 Expression

**Figure 4:**
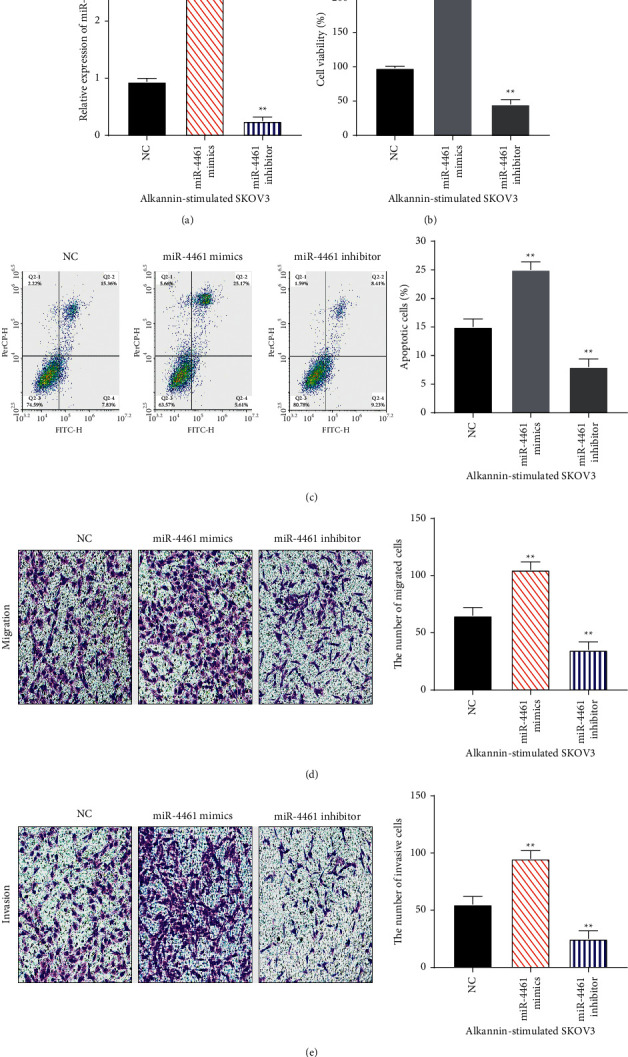
Alkannin restrains the progression of OC by decreasing miR-4461 expression. (a) miR-4461 expression was detected in alkannin-stimulated SKOV3 cells with miR-4461 mimics or inhibitor. (b, c) Cell proliferation and apoptosis were assessed in alkannin-stimulated SKOV3 cells with miR-4461 mimics or inhibitor. (d, e) Cell migration and invasion were detected in alkannin-stimulated SKOV3 cells with miR-4461 mimics or inhibitor (magnification, 200x). ^*∗∗*^*P* < 0.01.

**Figure 5:**
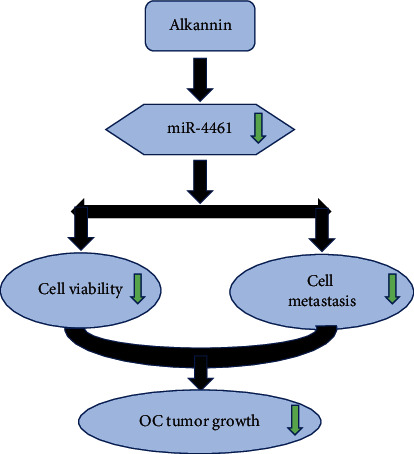
Alkannin inhibits cell viability and metastasis in OC by decreasing miR-4461 expression.

Finally, miR-4461 mimics and inhibitor were transfected into SKOV3 cells treated with alkannin. RT-qPCR showed that miR-4461 mimics increased its expression, while the miR-4461 inhibitor decreased its expression in SKOV3 cells treated with alkannin (*P* < 0.01, Figure 4(a)). Functionally, CCK-8 assay showed that miR-4461 overexpression promoted cell proliferation in alkannin-stimulated SKOV3 cells. However, downregulation of miR-4461 showed the opposite effect (*P* < 0.01, Figure 4(b)). At the same time, miR-4461 overexpression inhibited alkannin-stimulated SKOV3 cell apoptosis, while miR-4461 downregulation aggravated cell apoptosis (*P* < 0.01, Figure 4(c)). Besides, cell migration and invasion were both augmented by miR-4461 overexpression and inhibited by miR-4461 downregulation in alkannin-stimulated SKOV3 cells (*P* < 0.01, Figures 4(d) and 4(e)). The above results corroborate that alkannin inhibits the progression of OC by reducing miR-4461 expression (Figure 5).

## 4. Discussion

Commonly used drugs for OC are some chemotherapy drugs; the main drugs are cisplatin, carboplatin, paclitaxel, cyclophosphamide, and etoposide [19]. However, while chemotherapeutic drugs inhibit the proliferation of tumor cells, they also have a great killing effect on normal cells [20], which seriously affects their clinical application and efficacy. Therefore, the development of effective treatments for OC with fewer side effects is of great significance to women's health. After chemotherapy, the patient's body is in a state of qi and blood deficiency and is vulnerable to virus invasion [21]. TCM treatment starts from the whole and can improve the body's environment, improve immunity and disease resistance, and prevent tumor recurrence and metastasis [22]. In recent years, plant extracts have received widespread attention in cancer treatment due to their small side effects.

A large number of literature studies reported that plant extracts have a positive effect on inhibiting the viability of cancer cells. For instance, farrerol induced cancer cell death through ERK activation in SKOV3 cells [23]. Scutellarein induced apoptosis and inhibited the proliferation, migration, and invasion of OC cells by blocking EZH2/FOXO1 signaling [24]. Liu et al. reported that alkannin inhibited the viability, adhesion, invasion, and migration of human gastric cancer cell line MGC-803 through the toll-like receptor 2/nuclear factor-kappa B pathway [25]. Alkannin can also cause cell-cycle arrest and induce apoptosis by regulating the EGFR-NF signaling pathway in human epidermoid carcinoma A431 cells [26]. Similar to the above results, we also found that alkannin can inhibit cell proliferation and induce apoptosis in OC.

The metastasis of cancer cells plays an important role in the development of cancer. Many plant extracts have been found to suppress the metastasis of cancer cells. In this study, alkannin was found to inhibit cell migration, invasion, and EMT in OC. Similar to our results, Jang et al. proposed that alkannin prevents the migration and invasion of human breast cancer cells by inhibiting the activation of matrix metalloproteinase-9 [27]. The inhibitory effect of alkannin on cell metastasis has also been found in colorectal cancer [28]. More importantly, alkannin induced apoptosis and inhibited the migration of OC cells by inhibiting the phosphorylation of Src and FAK [29], which is consistent with our results. Different from the above studies, we found that alkannin plays an antitumor effect in OC by downregulating miR-4461 [18]. Moreover, miR-4461 can promote cell growth and metastasis in OC. This also confirmed the inhibitory effect of alkannin in the tumorigenesis of OC. However, our conclusion has not been verified by animal experiments. *In vivo* experiment will be conducted in the future.

## 5. Conclusion

In summary, this study shows that alkannin inhibits cell viability, metastasis, and EMT in OC by inhibiting miR-4461 expression. And alkannin can also prevent EMT in the progression of OC. However, the specific regulatory mechanism of alkannin in OC is still largely unknown. Therefore, more thorough experiments are still needed to corroborate this problem.

## Data Availability

The datasets used during the present study are available from the corresponding author upon reasonable request.
